# Antioxidant activity of mycelia methanolic extracts of endophytic fungi BvFV and BvFIX isolated from leaves of *Bauhinia variegata*

**DOI:** 10.3389/ffunb.2022.1048734

**Published:** 2022-12-02

**Authors:** Daniela Gurgel de Freitas Pires, Laíza Magalhães de Araújo, Pedro Góes Mesquita, Francisco de Assis Rocha Neves, Maria de Fátima Borin

**Affiliations:** Laboratory of Molecular Pharmacology, Department of Pharmacy, Health Sciences Faculty, University of Brasilia, Brasilia, Brazil

**Keywords:** endophytic fungus, skin aging, fibroblasts, antioxidant, PPAR, *Bauhinia variegata*

## Abstract

Endophytes are considered an essential source of natural products. Skin is the body’s largest organ; its primary function is the protection of other organs, and aging is one of the most relevant problems associated with this organ. UV radiation generates reactive oxygen species (ROS), which lead to skin degeneration and consequent aging. The main endogenous antioxidants that neutralize ROS are enzymatic antioxidants such as superoxide dismutase (SOD), catalase, glutathione peroxidase, and glutathione reductase, and non-enzymatic antioxidants, such as glutathione and α-tocopherol. Nuclear receptors are involved in molecular mechanisms that control the aging process, especially peroxisome proliferator-activated receptors (PPAR), which regulate the function and expression of genes that modulate the balance between matrix metalloproteinases (MMP) activity and the expression of collagen. Some natural compounds, such as polyphenols, can activate PPAR and reduce the activation of MMP and collagen degradation. In this work, the antioxidant activity of the mycelia methanolic extracts of two endophytic fungi isolated from leaves of *Bauhinia variegata*, named BvFV and BvFIX, their action as PPAR agonists, and their effect on the activity of antioxidant defense system enzymes were evaluated. The mycelia methanolic extract of BvFV showed a weak agonist effect on PPARβ/δ, a high capability to inhibit lipid peroxidation, increased catalase activity, and increased superoxide dismutase activity by approximately 64%. In contrast, BvFIX increased catalase activity and increased superoxide dismutase activity in a dose-dependent manner, with an increase of 49.62% ± 7.87%, 56.64% ± 12.27%, and 240.46% ± 26.11% at concentrations of 25 µg/mL, 50 µg/mL and 100 µg/mL, respectively, in human dermal fibroblasts submitted to oxidative stress. These results suggest that the metabolites of the mycelia of endophytic fungi studied are promising to act in the chemoprevention of skin aging.

## 1 Introduction

The skin is the largest organ in the human body, and its primary function is the protection of other organs (Nichols and Katiyar, 2010; Panich et al., 2016), and its extracellular matrix (ECM), with all its components in ideal amounts, allows the skin functions to be adequately fulfilled. This protective role is diminished with skin aging, an ordinary process involving genetic and environmental factors (Panich et al., 2016), and classified as intrinsic and extrinsic aging. Extrinsic aging is related to smoking, alcohol intake, nutrition, pollution, and ultraviolet (UV) radiation (Hwang et al., 2014; Mora Huertas et al., 2016). Epidemiological, clinical, and experimental studies have shown that UV radiation causes damage to the dermal connective tissue and is related to the development of various skin conditions, resulting in premature aging, called photoaging, and skin cancer (Nichols and Katiyar, 2010; Hwang et al., 2013; Briganti et al., 2014; Hwang et al., 2014; Kim, 2016; Mora Huertas et al., 2016; Panich et al., 2016). UV radiation can cause skin injuries with direct and indirect effects. The direct effects of UV radiation on DNA occur when nucleic acid absorbs UVB rays, resulting in a structural rearrangement of the nucleotides. The indirect effects are related to the tissues’ production of reactive oxygen species (ROS) (Nichols and Katiyar, 2010; Kim, 2016; Panich et al., 2016).

The skin has mechanisms for repairing oxidative damage, the antioxidant pathway (Panich et al., 2016), composed of enzymatic and non-enzymatic systems working to maintain the optimal level of ROS in cells. Examples of essential enzymatic antioxidants are superoxide dismutase (SOD), catalase, glutathione peroxidase (GPx), and glutathione reductase (GR), and among the non-enzymatic ascorbate, glutathione (GSH) and α-tocopherol (Nichols and Katiyar, 2010; Raut et al., 2012; Moukette et al., 2015; Panich et al., 2016; Kim, 2016). However, overexposure to UV radiation exceeds the body’s antioxidant defense capability, causing damage to lipids, proteins, and DNA that can be transmitted to daughter cells, leading to photoaging or even skin cancer (Nichols and Katiyar, 2010; Briganti et al., 2014; Panich et al., 2016; Kim, 2016). High levels of oxidative stress can also lead to lipid peroxidation by forming malondialdehyde (MDA), among other products. MDA combined with ROS causes more cell damage by interacting with cellular components, including DNA, causing genotoxicity (Pamplona, 2008; Das et al., 2015). The first step to protect against oxidation in the body is to eliminate the superoxide radical (O_2_·^-^), a step performed by SOD, which converts O_2_·^-^ into H_2_O_2_ (Panich et al., 2016; Zhang et al., 2016). Controlling the level of SOD activity and its expression contributes to the amount of SOD available and, consequently, to the level of ROS (Miao and St, 2009; Pérez et al., 2009; Panich et al., 2016). After SOD activity, catalase and GPx convert H_2_O_2_ formatted into H_2_O (Anjum et al., 2016; Zhang et al., 2016). In photoaging, there is a low catalase activity since repeated exposure to UVB rays suppresses the activity of SOD and catalase (Nichols and Katiyar, 2010; Kim, 2016). Lipid peroxidation induces rapid and significant depletion of intracellular GSH. Once GSH is depleted, the cells exhibit an intracellular change in the redox status and propagate a response to oxidative stress, followed by their production and the conversion of the oxidized glutathione back to GSH by GR. Thus, the system involved in GSH synthesis and recovery is crucial for maintaining the redox balance of the cell (Pamplona, 2008; Anjum et al., 2016).

The PPAR family is expressed in the skin in different cell types, and they are found in three isoforms, PPARα, PPARβ/δ, and PPARγ (Di-Poï et al., 2004; Jeon et al., 2015). PPARβ/δ is the most commonly found isoform in fibroblasts and, consequently, in the skin, and it has been shown that this isoform prevents degradation of type I and III collagen and secretion of matrix metalloproteinases type 1 (MMP1), the primary enzyme responsible for degradation of collagen, by inhibiting the generation of UVB-induced ROS in dermal fibroblasts (Jung et al., 2015).

Many phytochemicals have been investigated and demonstrated photoprotective activity on the skin. Polyphenols are the most promising since they have immunomodulatory, anti-inflammatory, and antioxidant activity (Nichols and Katiyar, 2010; Das et al., 2015). In addition, they can absorb the entire wavelength spectrum of UVB radiation and part of the UVA spectrum (Nichols and Katiyar, 2010; Lecci et al., 2021). Thus, polyphenols can act as a sunscreen, besides the chemoprevention, reducing inflammation, oxidative stress, and harmful effects on DNA caused by ultraviolet radiation on the skin (Nichols and Katiyar, 2010).

Endophytic microorganisms inhabit plant tissues throughout or part of their life cycle without causing them harm (Tan and Zou, 2001; Waqas et al., 2012; Sun et al., 2016). They are known as potential sources of natural compounds that have been shown to have various biological activities such as anticancer, antimicrobial, and antioxidant, which makes them of great pharmacological interest (Artanti et al., 2011; Liu et al., 2016). The search for secondary metabolites produced by endophytic fungi is becoming increasingly important as it provides an alternative strategy to alleviate the impact of the growing plant population required for the production of medicines, as well as for biodiversity and ecosystem preservation (Tan and Zou, 2001; Sun et al., 2016). *Bauhinia variegata* is a plant with antioxidant and antidiabetic activity, and the latest studies have attributed these activities to polyphenols and flavonoids (Farag et al., 2015). The ability of endophytes to produce host-like compounds makes endophytic fungi extracted from *B. variegata* become an important and interesting source of compounds, which may grow into a new approach to protection against photoaging (Tan and Zou, 2001; Zhou et al., 2010).

## 2 Material and Methods

### 2.1 Fungal submerged liquid culture

Two strains of endophytic fungi previously isolated from leaves of *Bauhinia variegata* were selected for this study. The fungi genetic sequences of identification were deposited in GenBank as BvFV (OP604490) and BvFIX (OP604493) (Mesquita PG, data submitted to publication). Five slants of Sabouraud Dextrose Agar (Himedia) of each fungus were previously cultured for seven days at 30°C. The mycelium and spores were harvested from the surface of the slants employing a stiff platinum loop and transferred to 50 mL of pre-fermentation medium described by Jackson et al. (Jackson et al., 1993) and incubated at 30°C and 150 rpm. After 48 h, these pre-cultures were transferred to an Erlenmeyer with 1000 mL of previously described fermentation medium (Jackson et al., 1993) and kept in the same conditions for 72 h.

### 2.2 Crude extracts production

After 72 h cultivating the fungi, the resulting culture medium was filtrated under vacuum. The mycelial was weighed and transferred to flasks containing 500 mL of methanol, where it remained in maceration for seven days to assurance the death of the fungi. After this, the macerated was filtrated in Whatman grade 1 filter paper under vacuum, and the methanol was evaporated in a rotary evaporator (Heidolph, Laborota 4010 - digital) at 40°C under vacuum to obtain the methanolic crude extracts.

### 2.3 Crude extracts fractionation

The methanolic crude extracts produced were solubilized in a hydroalcoholic solution (10 mL of methanol and 30 ml of distilled water) and fractionated by a liquid-liquid partition with hexane and ethyl acetate. Hexane and ethyl acetate fractions were dried in a rotary evaporator at 40°C under vacuum, and the hydroalcoholic fractions were dried by lyophilization (Liobras, Liofilizador L108). This fraction was denominated methanolic fraction. The fractions were weighed and dissolved in 1 mL of methanol.

### 2.4 Determination of total polyphenols content

The total polyphenols content of fractions of the extracts was determined by the Folin-Ciocalteu assay (Georgetti et al., 2006) with a few modifications. Briefly, 100 µL of the sample was mixed with 1.9 mL of water, 250 µL of Folin-Ciocalteu (Sigma-Aldrich) reagent, and 250 µL of 10% sodium carbonate solution. Gallic acid was used as standard, and total polyphenolic contents of samples were expressed as gallic acid equivalents per mg of extract (EAG/mg).

### 2.5 Determination of total flavonoids content

The total flavonoids content was determined using the aluminum chloride assay (Georgetti et al., 2006) with a few modifications. Briefly, 100 µL of extract solution was mixed with 1.9 mL of methanol and 500 µL of 5% aluminum chloride solution. The reaction mixture was homogenized in a vortex and incubated at room temperature for 30 min, and the absorbance was measured at 425 nm on a spectrophotometer UV/VIS (Shimadzu 06786). Quercetin was the standard, and the results were expressed as quercetin equivalent per mg of extract (EQ/mg).

### 2.6 Determination of antioxidant activity by DPPH· assay

The antioxidant activity was determined using the radical 2,2-diphenyl-1-picrylhydrazyl (DPPH·) radical scavenger assay (Chiang et al., 2012). Quercetin was used as standard, and the results were presented as a percentage of DPPH· reduction compared to the control, considering the absorbance of negative control as 100%. The IC_50_ value was determined for the extracts using eight different concentrations. A dose vs. response curve was drawn, and the IC_50_ value was determined in the GraphPad Prism 5 (GraphPad Software, Inc.) software.

### 2.7 Assessment of lipid peroxidation

The linoleic acid method was used to evaluate the inhibition of lipid peroxidation (Laokuldilok et al., 2011). Briefly, linoleic acid was emulsified with Tween 20 and then dissolved in 0.1 mol/L phosphate buffer pH 7, to which 100 µL of fungal extracts were added. This reaction was incubated for 8 h at 37°C, protected from light. After incubation, 100 µL of the emulsion were solubilized in 1 mL of methanol and 3 mL of 60% methanol solution. The absorbance was measured by spectrophotometry at 234 nm, in which the maximum absorbance of conjugated diene peroxide, a product of linoleic acid oxidation, occurs. Tocopherol at 100 µg/mL was used as a standard, and the percentage of inhibition of lipid peroxidation was calculated as previously described (Laokuldilok et al., 2011).

### 2.8 Treatment of cells for lipid peroxidation, superoxide dismutase, catalase, and reduced glutathione assays

Fibroblasts were plated at 3 x 10^5^ cells/well on 6-well plates in Dulbecco’s Modified Eagle Medium (DMEM, Sigma Aldrich) containing 10% fetal bovine serum (FBS, Gibco) and 100 U/mL penicillin and 100 mg/mL streptomycin, and added with fungal extracts solutions at concentrations of 25 µg/mL, 50 µg/mL, 100 µg/mL, or with the solvent used to solubilize the extracts only. Plates were incubated for 48 h at 37°C and 5% CO_2_. Next, cells were treated with 300 µM H_2_O_2_ for 1 h, and then, treatment was removed and cells incubated with DMEM culture medium under the same conditions described above for 48 h.

Cells were collected in phosphate-buffered saline (PBS) and centrifuged at 4000 rpm for 5 minutes at 4°C. The pellet was resuspended in ice-cold water and subjected to 3 cycles of freezing and thawing to obtain the cell lysate. Quantifying cell lysate proteins was performed using the Lowry method (Lowry et al., 1951) using the standard albumin serum bovine (Sigma-Aldrich).

### 2.9 Fibroblast and HeLa viabilities evaluation by MTT assay

Cytotoxicity of extracts was assessed according to the method of 3-(4,5-dimethyl-2-yl)-2,5-diphenyl tetrazolium bromide (MTT) (Mosmann, 1983). Briefly, 1x10^4^ cells (fibroblasts or HeLa) were plated in 96-well plates, treated with fungal extracts solutions at 6.25 µg/mL, 12.5 µg/mL, 25 µg/mL, 50 µg/mL and 100 µg/mL concentrations and incubated for 48 h (fibroblasts) or 24 h (HeLa) in an atmosphere of 5% CO_2_, 95% humidity at 37°C.

### 2.10 Transient transfection and treatment

Transient transfection was performed in HeLa cells using Lipofectamine^®^ 2000 (Invitrogen) to evaluate the agonist activity of the extracts to PPARγ, PPARα, and PPARβ/δ receptors. One day before transfection, 50.000 cells were seeded in 48-well plates with a medium free of antibiotics. Chimeric plasmids were used with one PPAR isoform and luciferase reporter plasmid driven by the GAL4 responsive element (Gal-Luc). Cells were treated with different fungal extracts or with 10^-7^ mol/L rosiglitazone (positive control for PPARγ), 10^-4^ mol/L bezafibrate (positive control for PPARα), 10^-3^ mol/L bezafibrate (positive control for PPARβ/δ), or the vehicle used for sample preparation (negative control), were placed 6 h after transfection and incubated for 22 h.

After this period, the medium was removed, and 100 µL of lysis buffer, 100X 100 mmol/L Tris/Triton pH 7.6, was added to each well. Then, 10 µL of cell lysate was transferred to a microtube, and 20 µL of luciferin was added to it for the luciferase oxidation reaction, measured in a luminometer (Promega, Glomax 20/20 Luminometer).

#### 2.10.1 Purification of PPARγ, PPARα and PPARβ/δ plasmids

The obtainment and purification of plasmids used in the transfection assay were carried out using Max Prep Qiagem – Tips method. The purity and concentration of the plasmids were determined by optical density at 260 nm and 280 nm in a NanoDrop spectrophotometer (Thermo Scientific 2000).

### 2.11 Valuation of lipid peroxidation in cells

The lipid peroxidation in cell lysate was evaluated using a thiobarbituric acid (TBA, Êxodo Científica) assay (Ohkawa et al., 1979). Thiobarbituric acid-reactant substances (TBARS) were measured fluorometrically at an excitation wavelength of 520 nm and an emission wavelength of 550 nm in a spectrofluorimeter (Beckman Coulter, DTX 800, Multimode Detector). MDA (Sigma-Aldrich) was used to construct a standard curve, and the cell lysate was submitted to a protein quantification assay (Lowry et al., 1951) to normalize the results obtained, which were expressed as nmol of MDA per mg of protein.

### 2.12 Evaluation of superoxide dismutase activity

SOD activity was measured in cell lysate by scavenging of SOD, using the xanthine/xanthine oxidase biochemical method (Weydert and Cullen, 2010) as a SOD generator and inhibiting nitroblue tetrazolium reduction as an activity indicator. Cells lysate samples were added to 700 µL of 50 mmol/L potassium phosphate buffer pH 7.8, containing 100 µmol/L EDTA, 60 µmol/L NBT, and 100 µmol/L xanthine. The final reaction volume was completed to 950 µL with 50 mmol/L potassium phosphate buffer pH 7.8, containing 100 µmol/L EDTA. Xanthine oxidase (0.075 U/mL) was added to samples, and then the absorbance was measured in a spectrophotometer in kinetic mode for 5 minutes at a wavelength of 560 nm. Cell lysate proteins were quantified using the Lowry method (Lowry et al., 1951) to normalize the results obtained.

### 2.13 Evaluation of catalase activity

Catalase activity was determined by monitoring the decomposition rate of H_2_O_2_ spectrophotometrically at a wavelength of 240 nm, according to the method described by Aebi, 1984 (Aebi, 1984). Cell lysate samples (50 μL) were added to 300 μL of 30 mmol/L H_2_O_2_ solution in 100 mmol/L sodium phosphate buffer, pH 7, and 650 μL of the same buffer. The reaction was monitored in the kinetic mode for 135 seconds at 240 nm. The molar extinction coefficient of 39.4 mmol/L.cm was used for H_2_O_2_, and results were expressed as a percentage of the unit of CAT per milligram of protein, considering the CAT activity of the non-stressed cells as 100%. One unit of the enzyme was defined as the amount necessary to decompose 1 mmol of H_2_O_2_ per minute at reaction conditions. Cell lysate proteins were quantified using the Lowry method (Lowry et al., 1951) to normalize the results obtained.

### 2.14 Evaluation of reduced glutathione

Determination of GSH was performed by the method of Hissin and Hilf (1976) with a few modifications. Briefly, for each milliliter of cell lysate, 200 µL of 30% trichloroacetic acid solution was added. Suspensions were centrifuged at 4000 rpm for 6 min at 4°C, and then the supernatant was collected and centrifuged at 10000 rpm for 10 min at 4°C. The supernatant was then used for GSH determination. Cell lysate proteins were quantified using the Lowry method (Lowry et al., 1951) to normalize the results obtained.

### 2.15 Statistical analysis

Statistical analyses were performed with GraphPad Prism 5. Data are expressed as mean ± standard error of the mean (S.E.M.). All assays were independently performed at least 3 times. The results were statistically evaluated using ANOVA one-way or two-way variance analysis followed by Dunnett’s test, Bonferroni or Turkey’s multiple comparison tests. Data were considered statistically significant when p < 0.05.

## 3 Results

In this study, the mycelial extract of two strains of endophytic fungi previously isolated from leaves of *Bauhinia variegata* collected in Brasilia, Brazil, BvFV and BvFIX, were evaluated for their antioxidant activity as possible skin protectors against oxidative stress.

### 3.1 Determination of total polyphenols and flavonoids contents and antioxidant activity of the extracts fractions

**Figure 1:**
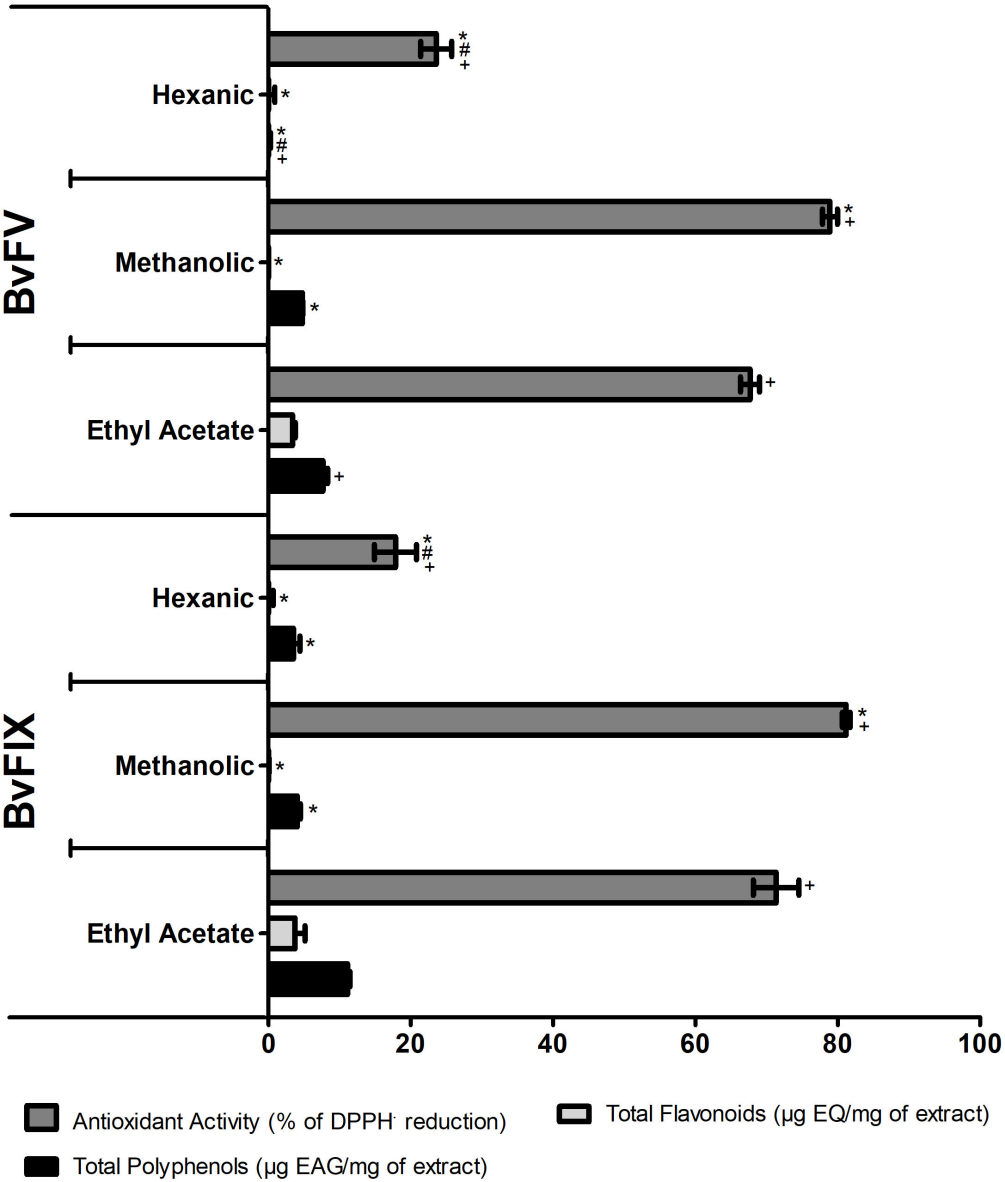
Total polyphenols and flavonoids contents and antioxidant activity of the fractions of the crude extracts of the BvFV and BvFIX mycelia. The total polyphenolic contents of samples were expressed as gallic acid equivalents per mg of extract (EAG/mg). The total flavonoid contents of samples were expressed as quercetin equivalent per mg of extract (EQ/mg). Antioxidant activity was a percentage of DPPH· reduction compared to the control. The results presented represent mean ± S.E.M. The statistical test applied was the analysis of variance (two-way ANOVA) followed by Bonferroni posttest. *, p < 0.05 vs. ethyl acetate fraction; #, p < 0.001 vs. methanolic fraction - comparisons among the different fractions of the same fungus. +, p < 0.001 - comparison between the fractions of the different fungi.

After producing the extracts of endophytic fungi, the first step of this work was to quantify the content of polyphenols and flavonoids, as they are secondary metabolites known by their antioxidant capability. The total polyphenols, the total flavonoids of the extracts fractions, and their antioxidant activity are shown in **Figure 1**. As expected, the most apolar extracts, the hexanic extracts, showed the lowest antioxidant activity and lowest polyphenols and flavonoids contents. The ethyl acetate fractions showed the highest content of polyphenols and flavonoids, and the methanolic fractions showed the highest antioxidant activity. The results suggest that polyphenols in the methanolic extracts are compounds other than flavonoids. Both fractions of the crude mycelial extracts of the fungi, methanolic and ethyl acetate, demonstrated results of polyphenols and antioxidant activity that made them of interest to continue the evaluations in other assays. Nevertheless, the production of methanolic extracts showed a better yield. For this work, the methanolic fractions of the crude extracts of the fungi mycelia were chosen to continue the experiments, and the following results refer to these fractions, named hereafter methanolic extracts.

### 3.2 Evaluation of antioxidant activity – determination of IC_50_

**Figure 2:**
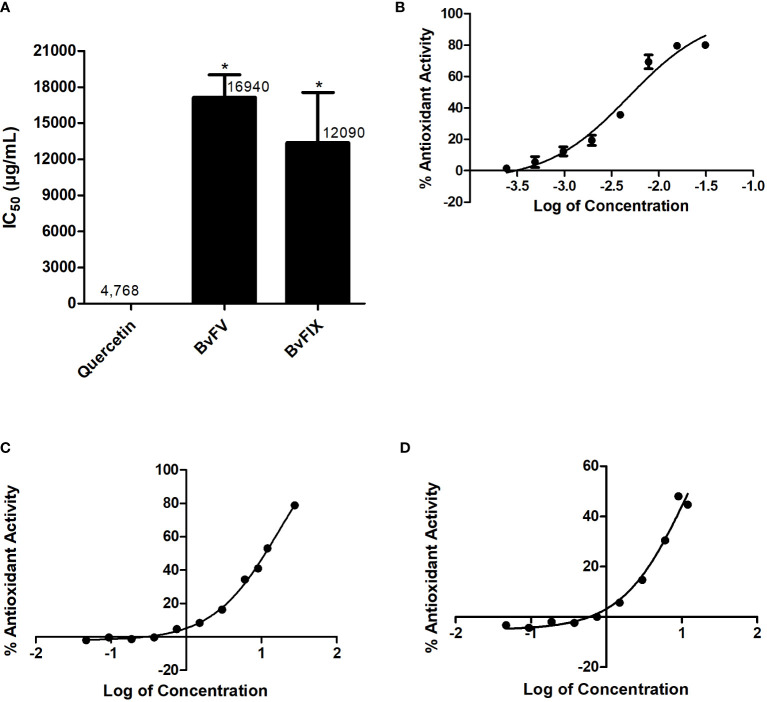
Antioxidant activity – determination of IC_50_. The IC_50_ value was determined for the extracts using eight different concentrations. A dose vs. response curve was drawn, and the IC_50_ value was determined in GraphPad Prism 5. **(A)** IC_50_, results represent mean ± S.E.M. *, p < 0.05 vs. quercetin, **(B)** Quercetin dose vs. response curve, **(C)** BvFV dose vs. response curve, and **(D)** BvFIX dose vs. response curve. The statistical test applied was the analysis of variance (one-way ANOVA) followed by Turkey’s multiple comparison tests.

The antioxidant activity of the extract fractions makes them good candidates for evaluating their possible protective action on cells exposed to oxidative stress. The antioxidant activity of the methanolic extracts was determined by DPPH· scavenger assay and expressed as IC_50_, the necessary concentration of extract to reduce 50% of DPPH· radical. Results are shown in **Figure 2**; the extract of the fungus BvFIX showed the lowest IC_50_ value (12.09 mg/mL) among the two fungi extracts.

### 3.3 Determination of the cytotoxic effect of the fungal extracts

**Figure 3:**
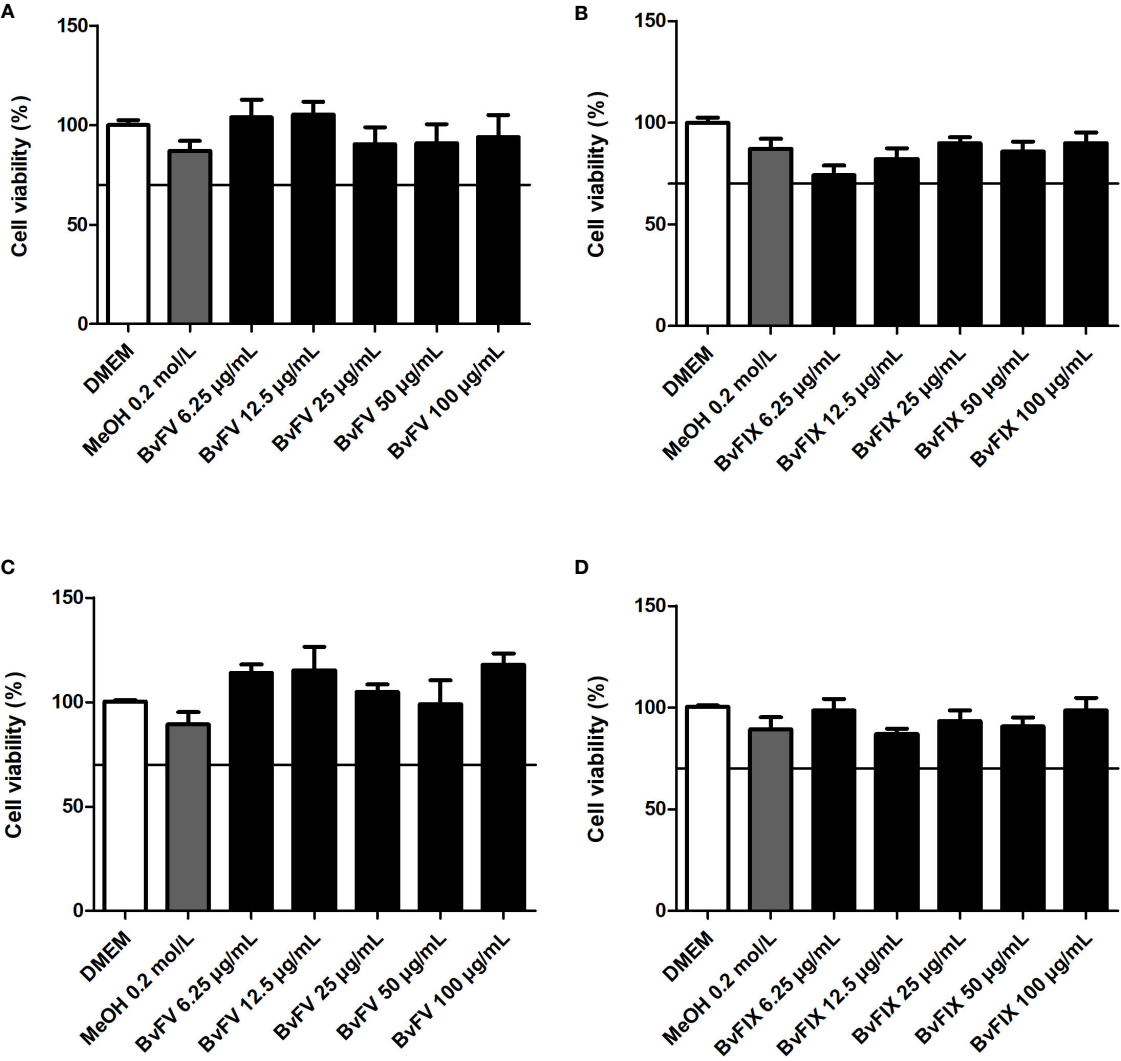
Evaluation of cytotoxicity of the fungal extracts. Human dermal fibroblasts were treated for 48 h with different concentrations of fungal extracts or with the solvent used to solubilize the samples (MeOH), **(A)** BvFV, and **(B)** BvFIX. HeLa cells were treated for 24 h with different concentrations of fungal extracts or with the solvent used to solubilize the samples (MeOH), **(C)** BvFV, and **(D)** BvFIX. The results presented represent mean ± S.E.M. in the percentage of viable cells.

Knowing that extracts had antioxidant activity, the MTT assay was performed to determine the cytotoxic effect of the fungi extracts and the concentration that could be used in treating the human dermal fibroblasts to evaluate catalase, SOD, and lipid peroxidation, as well as on the transfection assays on HeLa. The results obtained are shown in **Figure 3**. According to ISO-10993-5-2009 (International Organization for Standardization, 2009), the extract would have a cytotoxic potential if viability was reduced to less than 70% of the blank. Therefore, none of the extracts were toxic to human dermal fibroblasts or HeLa cells at the concentrations tested.

### 3.4 Evaluation of lipid peroxidation inhibition

**Figure 4:**
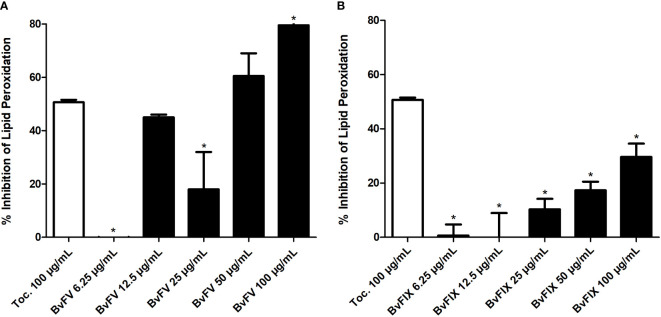
Assessment of lipid peroxidation. The linoleic acid method was used to evaluate the inhibition of lipid peroxidation. The results presented represent mean ± S.E.M. in the percentage of lipid peroxidation inhibition. **(A)** BvFV and **(B)** BvFIX. The statistical test applied was the analysis of variance (one-way ANOVA) followed by Dunnett’s test. *, p < 0.05 vs. tocopherol at 100 μg/mL.

ROS produced after UV exposure leads to lipid peroxidation, so, to further investigate the antioxidant activity of extracts, in this work, two assays were carried out to evaluate lipid peroxidation. The first one aimed to evaluate the inhibition of linoleic acid auto-oxidation. Methanolic extracts of the fungi demonstrated inhibitory activity of lipid peroxidation (**Figure 4**). The linoleic acid assay was used to determine the inhibition of lipid peroxidation, and α-tocopherol at 100 µg/mL concentration was used as a standard. Methanolic extract of BvFV at 100 µg/mL showed a higher capability to inhibit lipid peroxidation than α-tocopherol at the same concentration. On the other hand, methanolic extracts of BvFIX showed lower antioxidant activity in these assay conditions.

### 3.5 Evaluation of agonist activity to PPARγ, PPARα, and PPARβ/δ receptors

**Figure 5:**
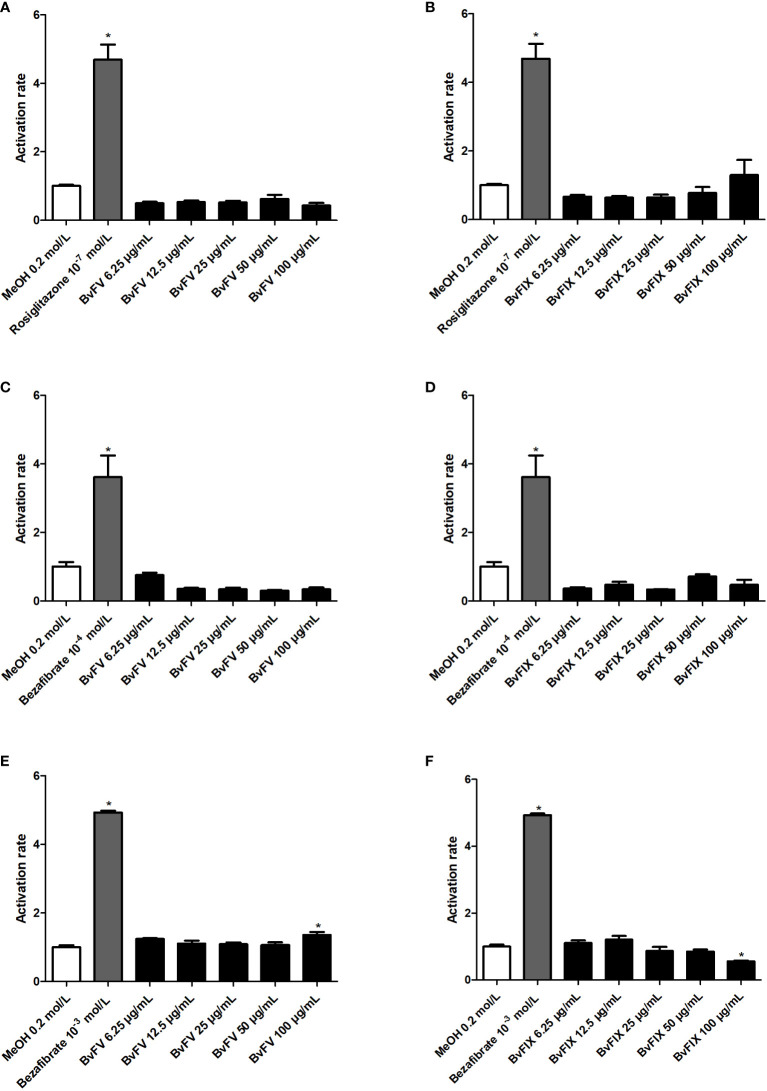
Evaluation of agonist activity of extracts against PPAR receptor. HeLa cells were treated for 22 h with different concentrations of extracts, with the solvent used to solubilize the samples (MeOH – negative control) only or with a PPAR agonist (positive control). The results presented represent the mean ± S.E.M of the PPAR activation rate. **(A)** PPARγ - BvFV, **(B)** PPARγ - BvFIX, **(C)** PPARα - BvFV, **(D)** PPARα - BvFIX, **(E)** PPARβ/δ - BvFV, and **(F)** PPARβ/δ - BvFIX. The statistical test applied was the analysis of variance (one-way ANOVA) followed by Dunnett’s test. *, p < 0.05 vs. MeOH (0.2 mol/L).

Evaluation of agonist activity of extracts to gamma, beta/delta, and alpha isoforms of PPAR receptor was carried out to evaluate its possible use as a chemoprotective agent in the skin by an activity exerted *via* activation of PPAR receptors. Results, shown in **Figure 5**, show that extracts did not show agonist activity at PPARα and PPARγ receptors, but the extract of BvFV at 100 µg/mL slightly activated the PPARβ/δ receptor. Extract of BvFIX at the same concentration (100 µg/mL) showed an activation rate of PPARβ/δ receptor significantly lower than that observed for the vehicle.

### 3.6 Evaluation of lipid peroxidation in cells

**Figure 6:**
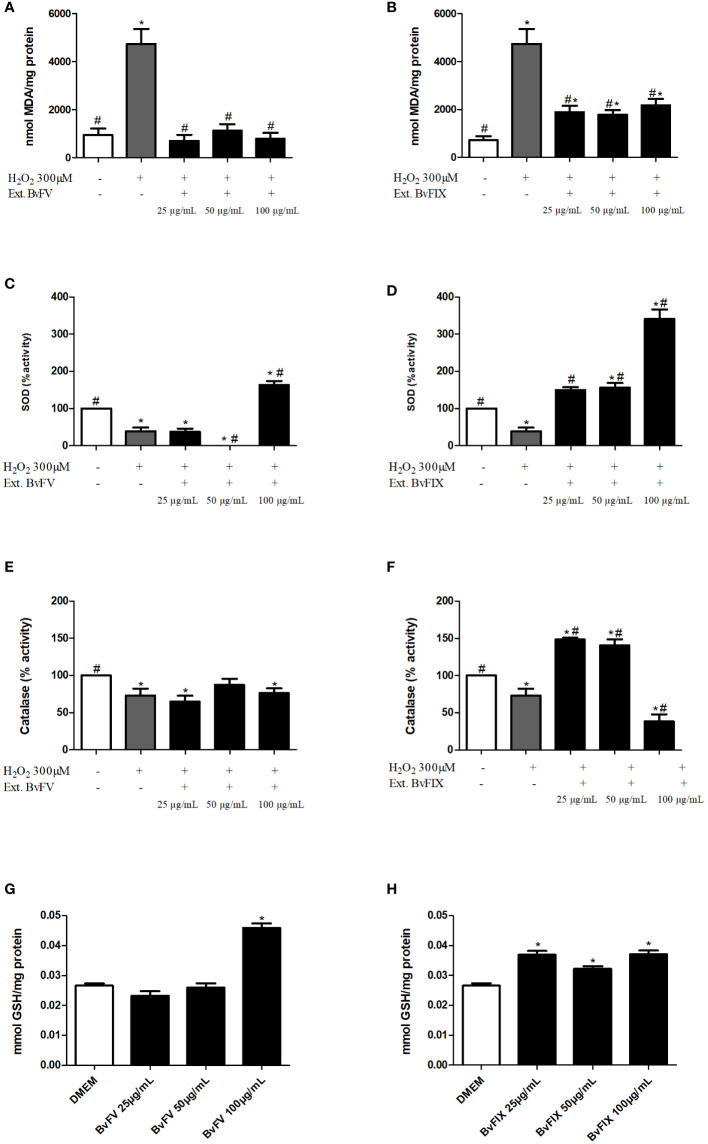
Assessment of antioxidant system in cells treated with fungal extracts. **(A-F)** Human dermal fibroblasts were treated for 48 h with different fungal extracts or only with the solvent used to solubilize the extracts (samples without extracts) and then inducted to oxidative stress by treatment with H_2_O_2_. **(A, B)** Results presented represent mean ± S.E.M of MDA concentration per mg of protein in cells treated or not with fungus extract: **(A)** BvFV, **(B)** BvFIX. **(C, D)** Results represent the mean ± S.E.M. of SOD enzyme activity: **(C)** BvFV, **(D)** BvFIX. **(E, F)** Results represent the mean ± S.E.M. of catalase enzyme activity: **(E)** BvFV. **(F)** BvFIX. **(G, H)** Human dermal fibroblasts were treated for 48 h with different concentrations of fungal extracts or only with the solvent used to solubilize the extracts (DMEM). Results represent the mean ± S.E.M. of GSH concentration: **(G)** BvFV, **(H)** BvFIX. The statistical test applied was the analysis of variance (one-way ANOVA) followed by Dunnett’s test. * and #, p < 0.05. * vs. DMEM or samples without extract, # vs. H_2_O_2_.

As mentioned before, two assays were carried out in this study to evaluate lipid peroxidation inhibition. The ability of fungal extracts to inhibit lipid peroxidation in fibroblasts was evaluated after the induction of oxidative stress in cells by treatment with H_2_O_2_. Results (**Figure 6**) showed that the methanolic fraction of BvFV extract inhibited lipid peroxidation, so MDA concentration remained similar to that found in cells that were not submitted to oxidative stress. This result suggests that the methanolic fraction of BvFV extract can protect fibroblasts from lipid peroxidation induced by oxidative stress, confirming the result observed in two other assays, DPPH, in which the extract had high antioxidant capacity, and inhibition of lipid peroxidation using the linoleic acid, in which its inhibition capacity was similar to that of the positive control α-tocopherol.

Methanolic fraction of BvFIX inhibited lipid peroxidation, reducing MDA concentration by approximately 60% compared to cells submitted only to treatment with H_2_O_2_. In this case, the result presented was also promising, because although the concentration found was higher than the average found for fibroblasts not subjected to oxidative stress, the extract of BvFIX was able to protect the cells from lipid peroxidation since it considerably reduced the concentration of MDA. This result is also compatible with those obtained for the DPPH assay, in which the extract showed good antioxidant activity, and in inhibition of lipid peroxidation by the linoleic acid assay, in which it showed an ability to inhibit lipid peroxidation.

### 3.7 Evaluation of superoxide dismutase activity

Superoxide dismutase is considered the skin’s first line of defense against ROS. Thus, to encompass more antioxidant activities of the extracts, SOD activity was evaluated by measuring inhibition of the reduction of nitroblue tetrazolium (NBT). Results were expressed as a percentage of SOD activity of the treated cells in comparison to the control, considering 100% of SOD activity for control cells that were not stressed with H_2_O_2_.

Treatment of human dermal fibroblasts with H_2_O_2_reduced SOD activity to 38.84% ± 10.05%, while treatment with BvFV fungus extract at 100 µg/mL concentration was managed to increase SOD activity by 63.99% ± 9.75%. The extract of BvFIX fungus led to an increase in SOD activity, in a dose-dependent manner, with an increase of 49.62% ± 7.87%, 56.64% ± 12.27%, and 240.46% ± 26.11%, in concentrations of 25 µg/mL, 50 µg/mL and 100 µg/mL, respectively (**Figure 6**).

### 3.8 Evaluation of catalase activity

Catalase is another critical enzyme to the human antioxidant system. Catalase activity was determined by spectrophotometrically monitoring the rate of H_2_O_2_ decomposition at a wavelength of 240 nm. The results were expressed as a percentage of catalase activity, with 100% activity attributed to control cells not induced to oxidative stress with H_2_O_2_.

Treatment of human dermal fibroblasts with H_2_O_2_ reduced catalase activity to 73.26% ± 9.18%. In contrast, treatment with methanolic extract of the fungus BvFV at concentrations of 25 µg/mL, 50 µg/mL and 100 µg/mL resulted, respectively, in the 64.71% ± 8.23%, 87.26% ± 8.44% and 76.22% ± 6.33% of catalase activity in comparison to control. Methanolic extract of mycelia of BvFIX caused an increase in catalase activity at concentrations of 25 µg/mL and 50 µg/mL, showing activity of 148.41% ± 2.39% and 140.71% ± 8.28%, respectively. At 100 µg/mL concentration, the activity was reduced to 38.24% ± 9.51%. Graphs in **Figure 6** represent these results.

### 3.9 Evaluation of reduced glutathione levels

Reduced glutathione levels represent a way to check cells’ redox status. Treatment of human dermal fibroblasts with methanolic extract of BvFV at a concentration of 100 µg/mL increased the concentration of GSH by 72.53 ± 5.80% compared to control, whereas treatment with methanolic extract of BvFIX at concentrations of 25 µg/mL, 50 µg/mL and 100 µg/mL it was able to increase by 38.83 ± 4.79%; 21.06 ± 3.59% and 39.38 ± 4.76% the reduced glutathione levels of the cells (**Figure 6**).

## 4 Discussion

Aging is a natural process that occurs in all animal species. It leads to many harmful changes, increasing the likelihood of undergoing degenerative processes (Pamplona, 2008). Skin aging is a complex biological process that occurs through the induction of intrinsic and extrinsic factors that result from the passage of time or action of environmental factors, such as radiation, mainly UV radiation (Chiang et al., 2012; Hwang et al., 2014; Jung et al., 2015; Mora Huertas et al., 2016; Panich et al., 2016). Search for immunomodulatory, anti-inflammatory, and antioxidant mechanisms constitute photoprotective strategies. Many antioxidant agents are phytochemical derivatives, and polyphenols are one of the main metabolites studied because they have these functions (Nadim et al., 2014; Bosch et al., 2015).

Crude methanolic extracts of the two endophytic fungi studied are capable of producing polyphenolic compounds. The polyphenol content found in the extracts evaluated in this study was also higher than that presented by the extract of *Michelia alba* leaves (2.7 ± 0.2 µg EAG/mg), which has protective activity in human dermal fibroblasts exposed to ultraviolet radiation by attenuating the expression of MMP-1, MMP-3 and MMP-9 promoting recovery of collagen synthesis (Chiang et al., 2012).

In this work, two assays were carried out to evaluate lipid peroxidation. The first one aimed to evaluate the inhibition of linoleic acid auto-oxidation. Results (**Figure 4**) showed that the methanolic fraction of BvFV crude extract, at concentrations of 12.5 µg/mL and 50 µg/mL, showed activity similar to α-tocopherol, positive control used at a concentration of 100 µg/mL, and the same concentration (100 µg/mL) of the extract of BvFV showed activity 79.50 ± 6.50% higher than α-tocopherol. Analyzing the DPPH· assay can be observed that this same extract showed high antioxidant activity. Lipid peroxidation assay in cells confirms the antioxidant activity of BvFV extract since the concentration of MDA in fibroblasts treated with the extract and stressed with H_2_O_2_ was statistically similar to that of MDA in cells that were not stressed with H_2_O_2_ (**Figure 6**). In the case of the extract of BvFIX, the three concentrations tested were able to reduce the concentration of MDA by more than 50% compared to cells submitted only to treatment with H_2_O_2_ (**Figure 6**), showing that it also presents promising results as an antioxidant.

When comparing with the literature, it is observed that both extracts are promising in the inhibition of lipid peroxidation since 50 µg/mL of hydroalcoholic extract produced from the bark of the stem of *Saraca indica*, which has antioxidant activity and the ability to reduce cytotoxicity and genotoxicity induced by irradiation with X-rays, was able to reduce MDA concentration after X-rays exposure by 1,5-fold (Das et al., 2015), and methanolic extracts of BvFV and BvFIX at the same concentration reduced MDA by approximately 4 and 2.5-fold, respectively, after induction of oxidative stress. A study by Perez et al., 2009, showed that the lifespan of mice lacking the sod1 gene was reduced compared to wild-type mice. The authors observed that levels of oxidative damage observed in the sod1^-/-^ mice were much higher than that commonly observed in tissues from old mice (Pérez et al., 2009).

The results of this work showed that BvFV extract increases SOD activity. The BvFIX extract increased in SOD activity, which was dose-dependent compared to enzyme activity in cells that did not undergo oxidative stress (**Figure 6**). When comparing results obtained with 50 µg/mL of hydroalcoholic extract produced from the bark of *Saraca indica*, which was able to increase SOD activity by 1.3-fold after exposure to X-rays (Das et al., 2015), BvFIX metabolites can be considered promising in reducing premature skin aging, since the methanolic fraction of BvFIX extract at same concentration increased SOD activity by 3.6-fold after induction of oxidative stress. H_2_O_2_ produced by SOD activity is broken down into water and oxygen by catalase (Pérez et al., 2009; Kim, 2016). The methanolic fraction of BvFIX extract increased catalase activity, even among cells that did not undergo oxidative stress (**Figure 6**).

ROS produced after UV exposure leads to a rapid and significant depletion of intracellular GSH. Thus, the system involved in synthesizing and recovering GSH is necessary to maintain cells’ redox balance (Pamplona, 2008; Zapatero-Solana et al., 2014). In this work, GSH was quantified, and methanolic fractions of the extracts increased the concentration of GSH in fibroblasts after being subjected to oxidative stress (**Figure 6**). When compared with VC-3LG, an AsA derivative that reinforces the intracellular antioxidant system, it can be observed that it is capable of increasing the concentration of GSH by approximately 25% and 50% in keratinocytes of human epidermis not subjected to oxidative stress in concentrations of 3 µM and 10 µM, respectively (Katsuyama et al., 2016). Therefore, BvFV and BvFIX fungi extracts may also be promising in their ability to strengthen the intracellular antioxidant system.

Antioxidant enzymes may have their expression increased by PPARs action (Briganti et al., 2014; Shin et al., 2016). In this study, the agonist activity of methanolic fractions of the extracts to the PPARα, PPARβ/δ, and PPARγ receptors was evaluated. The results indicated that BvFV extract at 100 μg/mL concentration slightly activates the PPARβ/δ receptor (**Figure 5**). In conclusion, the results of this work showed that the methanolic fraction of BvFV and BvFIX mycelia extracts have antioxidant activity and the ability to increase the activity of the main antioxidant enzymes of the dermis, even after induction of oxidative stress with oxygen peroxide, suggesting their application in chemoprevention of skin aging. The maceration of the fungi mycelia in methanol for seven days was chosen as the extractive method of the metabolites to ensure the death of the fungi. New conditions of extractions must be conducted to optimize the extractive process and evaluate the composition and activity of the metabolites extracted. Also, further studies should be conducted to confirm and investigate these extracts’ activity in protecting against photoaging.

## Data availability statement

The raw data supporting the conclusions of this article will be made available by the authors upon request.

## Author contributions

DF performed the experiments, analysed the data, and wrote the manuscript with guidance from MB. LA contributed to the design and execution of the experiments. PM isolated and identified the fungi used in this work. FN coordinated the master project. MB conceived and designed and coordinated this study. All authors contributed to the manuscript and approved the submitted version.

## Funding

This work was supported by grants from the Federal District Research Support Foundation (FAPDF - process 0193.000977/2015), the National Council for Scientific and Technological Development (CNPq), the Ministry of Science, Technology, Innovation and Communications (MCTIC) and the National Fund for Scientific and Technological Development (FNDCT - process 407851/2013-5 – Pro-Centro-Oeste Research and Postgraduate Network). This study was financed in part by the Coordenação de Aperfeiçoamento de Pessoal de Nível Superior – Brasil (CAPES) – Finance Code 001.

## Conflict of interest

The authors declare that the research was conducted in the absence of any commercial or financial relationships that could be construed as a potential conflict of interest.

## Publisher’s note

All claims expressed in this article are solely those of the authors and do not necessarily represent those of their affiliated organizations, or those of the publisher, the editors and the reviewers. Any product that may be evaluated in this article, or claim that may be made by its manufacturer, is not guaranteed or endorsed by the publisher.
